# The American Association of Obstetric and Gynecologists

**Published:** 1888-08

**Authors:** 


THE AMERICAN ASSOCIATION OF OBSTETRICS AND GYNECOLOGISTS will be held in Washington, D. C., September 18, 19 and 20, 1888. A discussion will take place on "Extra Uterine Pregnancy," and other interesting subjects. Many important papers have already been announced to be read by prominent men in these specialties. Mr. Lawson Tate, Dr. Frank Townsend, Dr. E. E. Montgomery, Dr. Charles Reid, Dr. A. Vanderveer and others will participate in the discussions. The address of the President, Dr. Wm. H. Taylor, of Cincinnati, is looked forward to with interest.

